# A Study of Mobile Medical App User Satisfaction Incorporating Theme Analysis and Review Sentiment Tendencies

**DOI:** 10.3390/ijerph19127466

**Published:** 2022-06-17

**Authors:** Yunkai Zhai, Xin Song, Yajun Chen, Wei Lu

**Affiliations:** 1 School of Management Engineering, Zhengzhou University, Zhengzhou 450001, China; zhaiyunkai@zzu.edu.cn (Y.Z.); 202012302015027@gs.zzu.edu.cn (X.S.); 202012302015023@gs.zzu.edu.cn (Y.C.); 2 National Engineering Laboratory for Internet Medical Systems and Applications, Zhengzhou 450052, China

**Keywords:** online reviews, satisfaction evaluation, mobile medical apps, fuzzy comprehensive evaluation, LDA topic model

## Abstract

Mobile medicine plays a significant role in optimizing medical resource allocation, improving medical efficiency, etc. Identifying and analyzing user concern elements from active online reviews can help to improve service quality and enhance product competitiveness in a targeted manner. Based on the latent Dirichlet allocation (LDA) topic model, this study carries out a topic-clustering analysis of users’ online comments and builds an evaluation index system of mobile medical users’ satisfaction by using grounded theory. After that, the evaluation information of users is obtained through an emotional analysis of online comments. Then, in order to fully consider the uncertainty of decision makers’ evaluations, rough number theory and the fuzzy comprehensive evaluation method are used to confirm the conclusions of experts and indicators and to evaluate the satisfaction of mobile medical users. The empirical results show that users are satisfied with the service quality and content quality of mobile medical apps, and less satisfied with the management and technology qualities. Therefore, this paper puts forward corresponding countermeasures from the aspects of strengthening safety supervision, strengthening scientific research, strengthening information audit, attaching importance to service quality management and strengthening doctors’ sense of gain. This study uses text mining for index extraction and satisfaction analysis of online reviews to quantitatively evaluate user satisfaction with mobile medical apps, providing a reference for the improvement of mobile medical apps. However, there are still certain shortcomings in the current study, and subsequent studies can screen false reviews for a deeper study of online review information.

## 1. Introduction

In recent years, the level of medical quality has increased year by year, but its spatial inequality has become increasingly prominent. There is a huge accessibility gap between the developed areas and the underdeveloped areas in first-tier cities and provincial capitals, such as Beijing and Shanghai, and with the decline in urban agglomeration, the degree of the gap is increasing. In particular, the outbreak of the coronavirus pandemic in 2020 led to an increase in the problem of being able to see a doctor in less developed regions, and the traditional medical model is no longer sufficient to meet the needs of the public. As the information technology and digital revolution continues, medical services have entered the mobile era [1]; mobile medical apps, as an important means of online consultation, are third-party medical and health models based on mobile communication technology that provide medical and health-related content for the purpose of promoting diagnosis and disease prevention [2]. Mobile medical apps not only provide clinicians with easy access to medical knowledge and patient data [3], but also improve the accessibility of medical care for patients, optimize the allocation of quality medical resources to some extent, and play an important role in improving the efficiency and convenience of medical care, thereby improving the health status of the population and alleviating conflict between doctors and patients [4]. At present, China’s mobile medical industry is developing rapidly, and the mobile medical app “Good Doctor Online” provides a new method of medical consultation for the majority of users. However, the development of mobile medical usage in China is still in a primary stage, and there are different quality problems with different major applications. Maintaining and improving users’ satisfaction with mobile medical apps and speeding up the development of mobile medical apps are significant problems in the development of mobile medicine in China [5]. A scientific and comprehensive evaluation system for this new model of health care services is needed to measure its operational effectiveness, in order to better provide quality services that meet the needs of the people and promote further improvement in the quality of public health services and the health of the people. This study on the satisfaction evaluation of mobile medical apps is helpful in reviewing and reflecting on the usage of mobile medical apps and in promoting the sustainable development of mobile medical.

### 1.1. User Satisfaction

User satisfaction refers to the psychological feeling of satisfaction or disappointment formed by users after comparing the perceived results of products or services with their own expectations [6]. For example, user satisfaction with mobile medical apps directly reflects the degree of users’ recognition of mobile medical care; it is also an important indicator of the effectiveness and development of mobile medical usage. Scholars mainly obtain information through questionnaires, interviews, and other forms to evaluate user satisfaction. This method of information acquisition requires the design of some questions in advance, a process that is highly subjective. Its results are strongly dependent on the preset questionnaire, which limits the evaluations by users to the evaluated subjects. In addition, due to the constraints of time, costs, manpower, and material resources, such methods can only collect a small amount of data within a certain time range, and the reliability of the results fluctuates greatly according to the scale of the investigation [7,8].With the development of information technology, such as Web 2.0, e-commerce, and social media, online reviews have become important information resources for influencing consumer decisions and product sales [9]. Online reviews have the advantages of large data volume and low collection costs [10], and they are able to contain more complex contents than those of other methods of review, better reflecting users’ subjective feelings from multiple dimensions and helping service providers to understand users’ needs and feelings in making operational decisions.

### 1.2. Selection of Indicators and Construction of Evaluation System

The selection of indicators and the construction of an evaluation system are the key steps in the evaluation of user satisfaction with mobile medical apps. Currently, some scholars choose to use online ratings from online reviews to study customer satisfaction; however, online ratings alone cannot result in a full understanding of customer evaluation information or provide sufficient evidence for improving customer satisfaction [11]. In contrast, online reviews involve users’ active participation rather than passive responses, and users’ active sharing of their experiences and their own feelings can better reflect their true perceptions and needs. Accordingly, more and more scholars have begun to pay attention to the potential information contained in online reviews. With the development of text mining technology, such as topic extraction and sentiment analysis, there are many methods suitable for handling large amounts of unstructured data, such as online comments.

In 2003, Blei et al. [12] proposed latent Dirichlet allocation (LDA), which is the most widely used topic extraction model and has been developed in the field of text analysis. LDA is an unsupervised learning method that can effectively mine and discover potential semantic topics and their probability distributions in text data. It has shown advantages in large-scale text mining and information processing. Scholars have started to apply LDA topic models to the mining of satisfaction-influencing factors. For example, Guo et al. [13] used the LDA model to extract 19 satisfaction dimensions of visitors’ evaluations of hotels based on online reviews. Srinivas et al. [14] pointed out that 95% of students rely on positive word-of-mouth in choosing a university; they extracted topics based on the LDA topic model for students’ online reviews and used them as key factors that influence students’ satisfaction with universities to provide decision guidance for school leaders. Gao et al. [15] used the improved topic model to conduct topic modeling for online medical reviews and to mine patients’ concerns about medical services. Tian et al. [16] used the LDA topic model to mine the content of online teaching evaluations, which in turn led to a demand index system for online teaching.

However, based on the LDA topic model alone, it is not yet possible to scientifically construct a user satisfaction evaluation index system for mobile medical apps. Only by systematically identifying and analyzing elements of user concern can we understand the key elements affecting user satisfaction at different levels. Grounded theory is able to propose new concepts and theoretical frameworks, based on the existing research materials, by way of extracting, coding, and testing. Therefore, in order for satisfaction evaluation to be more scientific, this study combines grounded theory with LDA topic mining to construct an index system.

### 1.3. Sentiment Analysis

In a satisfaction evaluation, users’ opinions and attitudes should be observed. Therefore, after constructing an evaluation index system, it is necessary to understand the extent of users’ evaluations of services so that service providers can accurately understand users’ needs and attitudes and make decisions. Sentiment analysis can determine whether a user’s view of a service is positive or negative from the text; it has been widely used in customer satisfaction measurement based on online reviews. For example, after analyzing the emotional tendencies of consumers’ online reviews, Zhang et al. [17] explored the keywords in negative consumer reviews and analyzed the outstanding problems with the relevant products, as reflected in the reviews, so as to improve product features and service quality. Liu et al. [18] used theme discovery and sentiment analysis to identify information expressed in comments on medical websites. Xiang et al. [19] used a feature rule approach, with topic mining and sentiment analysis of the content of medical users’ online reviews of hospitals. In these ways, sentiment analysis can identify the extent of users’ sentiments in online reviews of mobile medicine and transform the text information into numbers that indicate the level of user satisfaction to facilitate a subsequent quantitative evaluation.

### 1.4. Satisfaction Evaluation Method

The limited nature of individual perceptions and the complexity of assessment metrics result in a wide range of uncertainties for decision makers in the decision-making process. Currently, user satisfaction evaluations are usually analyzed quantitatively by way of subjective evaluations, with less consideration given to ambiguity and uncertainty in the information evaluated by decision makers. Compared with traditional evaluation methods, the fuzzy comprehensive evaluation method can quantify the inherent fuzziness of users’ emotions in a scientific and reasonable manner through numerical forms; this method has certain advantages in converting a qualitative evaluation into a quantitative evaluation. For example, Geng et al. [20] constructed an evaluation index system for tourist satisfaction in regard to ancient village scenic spots and used fuzzy comprehensive evaluation to measure tourist satisfaction in the scenic spot of Imperial City Xiangfu; the evaluation efficiency and rationality of this method was relatively high. On the other hand, the weight of expert opinions and evaluation indices also affect decision results, and most existing user satisfaction evaluations only consider expert opinions or evaluation indices individually, which greatly reduces the accuracy of evaluation results. There are many methods to deal with this problem, such as fuzzy sets [21], triangular fuzzy numbers [22], evidential reasoning [23], and intuitive fuzzy numbers [24]. However, these methods are strongly influenced by a priori knowledge. Rough number is a method of quantifying expert cognition, using rough number intervals to portray the fuzziness and uncertainty of expert evaluation information without a priori knowledge components such as an affiliation function or a utility function; this method determines group preferences based entirely on expert evaluation information.

The present study uses the method of text mining to subject-mine user reviews of mobile medical apps, summarizing user attention elements according to grounded theory so as to construct a user satisfaction evaluation system for mobile medical apps. Then, by analyzing the sentiment tendency quantified in the review, the evaluation data set of users may be obtained. On this basis, the rough number theory and the fuzzy comprehensive evaluation method are integrated to evaluate and analyze user satisfaction, and the influence of fuzziness and uncertainty on decision result is fully considered.

## 2. Overall Architecture of the Evaluation Model

Based on users’ online review data, this study conducts in-depth mining and analysis of user satisfaction with the help of text mining, topic analysis, grounded theory, sentiment analysis, and fuzzy evaluation, and then conducts a fuzzy comprehensive evaluation of user satisfaction on the basis of considering experts and attributing weights, as well as information fuzziness. The study identifies attributes that users pay attention to and analyzes the mechanisms of the influencing factors of user satisfaction, and provides guidance and suggestions for enhancing user satisfaction. It also provides directions to enhance user satisfaction with and service quality of mobile medical apps, as well as guidance suggestions for improving the efficiency and effectiveness of mobile medical apps’ operation and management.

**Figure 1:**
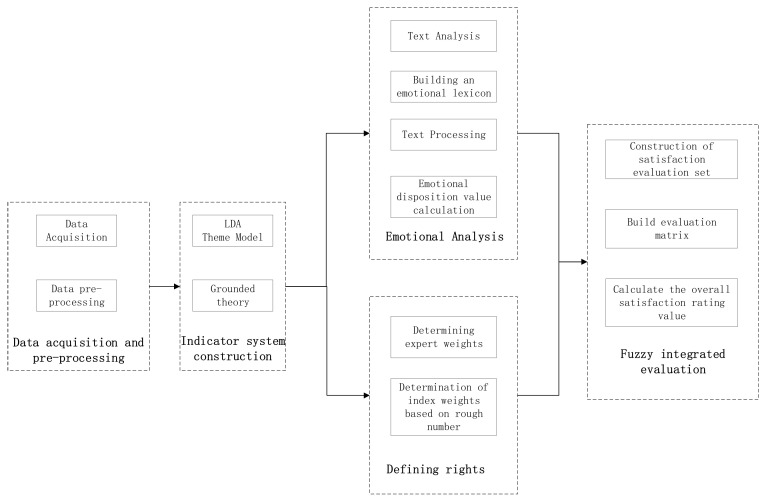
General framework diagram.

The overall structure of the model is shown in Figure 1, including five steps: data collection and pre-processing, evaluation index system construction, sentiment quantification analysis, determination of the weight of evaluation indices, and fuzzy comprehensive evaluation of satisfaction. In the first step, data collection and pre-processing, the collected data are cleaned, Chinese word separation is carried out, and deactivated words are removed. In the second step, constructing evaluation index system, the LDA topic model is used for topic training, identifying user concern indexes, and constructing a user satisfaction evaluation index system by summarizing via grounded theory. In the third step, obtaining the evaluation set based on sentiment analysis, after analyzing the sentiment tendency of text comments, the texts are classified and a sentiment dictionary is built to process the degree of adverbs, negatives, and exclamations, and then the sentiment is quantified and the users’ evaluation value for each index is obtained. In the fourth step, determining the weight, weights are assigned to experts according to importance, and the weights of evaluation indices are determined in combination with rough number theory. In the fifth step, the fuzzy comprehensive evaluation of satisfaction is used to conduct a comprehensive measurement of user satisfaction with mobile medical apps.in order to fully consider the fuzziness and uncertainty of user evaluation information in the decision-making process.

## 3. Mobile Medical App User Satisfaction Evaluation Model

### 3.1. Data Acquisition and Pre-Processing

Online reviews are an important way to obtain users’ feedback on products and users. With the gradual improvements in text analysis technology, the value of online review data has been effectively utilized. Therefore, this study collects online comment data with the help of Python web crawler technology, including information such as user nicknames, comment time, and comment content. Since the directly crawled online comment data are relatively rough and raw, they need to be pre-processed before text mining. First, in order to obtain reliable corpus information, data cleaning, including the removal of irrelevant content, standardizing the format, and removing abnormal and duplicate data, was performed to establish a reliable corpus source. Second, in order to correctly identify and classify the proprietary vocabulary, a user dictionary of this study was constructed. Then, in order to extract the topic features of the text, the comment text was divided into Chinese words and the deactivated words were removed. We used the Jieba package in Python for word separation and filtered out words and symbols with no practical meaning, such as “the” and “Hello”, using the deactivation word list of Harbin Engineering University.

### 3.2. Identification of Factors Influencing Mobile Medical APP User Satisfaction

Identifying the key factors influencing user satisfaction from online reviews is an important task in satisfaction evaluation. There are several commonly used subject extraction techniques, such as the extraction of subject terms based on term frequency- inverse document frequency (TF-IDF), the Textrank algorithm for web-based recommendation systems, and PageRank (the unsupervised topic extraction algorithm). However, these methods are more suitable for scenarios with a high number of noisy words and a single form of output, and they do not reveal the underlying relationships between the subject words and the original text.

Compared with the above-mentioned text processing methods, the LDA topic model can reduce the dimensionality of the text representation by a semantic analysis of large-scale text, thus extracting more valuable potential topics and revealing the intrinsic relationship between different topic words to a certain extent. Therefore, this study used the LDA topic model to topic-mine the online review texts of mobile medical app users, so as to identify the attributes that users pay attention to in mobile medical apps.

**Figure 2:**
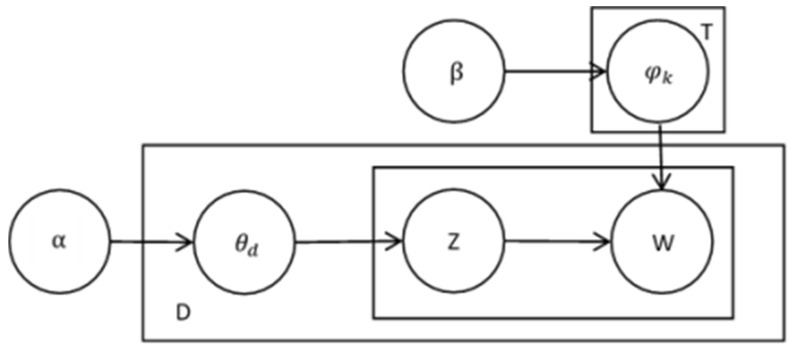
LDA probability model diagram.

LDA is a probabilistic growth model constructed for discrete data sets. It is a three-layer Bayesian model that associates words and documents through potential topics. Itis a probabilistic model with a 3-layer structure of documents, topics, and words. In the LDA topic probability, the comment text topic probability distribution $\theta_{d}$ is generated by sampling from the Dirichlet distribution with parameter α, while the word distribution of a topic is generated from parameter $\varphi_{k}$ by sampling from the Dirichlet distribution with parameter β. In the context of this study, the steps for generating text based on online reviews of mobile medical LDA topic probabilities are as follows: (1) Extract the probability distribution of the “document-topic”, i.e., distribution relation $\theta_{d}$, for the probability of sampling text topics from the Dirichlet distribution with parameter α; (2) extract the subject term $\varphi_{k}$ from the polynomial of the probability distribution of the “document-topic” of β; (3) draw subjects from the Dirichlet distribution with parameter β in relation to the word probability distribution, i.e., parameter $\varphi_{k}$; and (4) extract the topic words $W_{\mathrm{dn}}$ from the polynomial of the probability distribution of “topic-word” of $\varphi_{k}$. A Bayesian network graph was used to represent the way the LDA topic model generates a text, as shown in Figure 2.

Grounded theory starts from actual observation, summarizes on the basis of empirical information, and then constructs relevant social theories from the bottom up. Therefore, this study summarizes user attention attributes based on the research method of grounded theory, identifies the main factors affecting user satisfaction and the evaluation dimensions to which they belong, and then constructs a user satisfaction evaluation index system that can better fit the actual situation with a certain scientificity.

### 3.3. Integration of Evaluation Information Based on Sentiment Disposition Analysis

First, according to Chinese wording habits, the comment statements were assigned to the corresponding categories of evaluation indicators at each level, and the comments that did not belong to the evaluation system were deleted. Since irregular spoken comments are common (e.g., the comment sentence “Very serious answers, doctors are also very professional, and the responses are very timely“), they may contain evaluation indicators belonging to two different categories; therefore, the comment sentence would be classified into two categories. 

**Figure 3:**
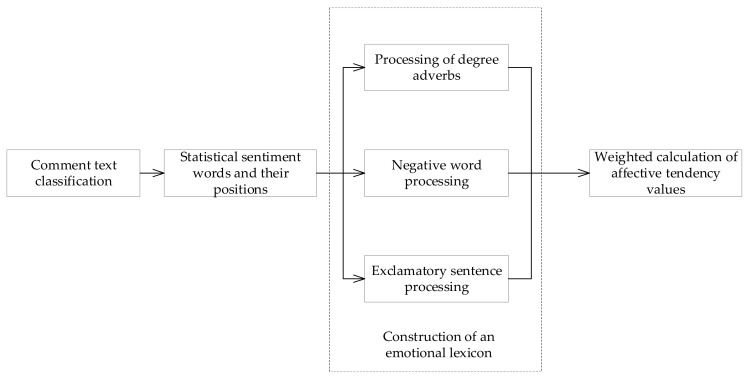
Emotional analysis.

Second, the sentiment tendency value of user evaluation indicators was calculated based on text sentiment analysis. Text sentiment analysis is divided into two alternatives based on machine learning or lexicon, and considering the short text and sentence-level characteristics in an online review text, it is usually more suitable to adopt the lexicon approach. Therefore, for the emotional characteristics of users’ online reviews of mobile medical apps, the corresponding emotional lexicon was constructed based on two types of emotional words, positive and negative, and the intensity of emotional polarity was modified by assigning different weights to degree adverbs, while the important influence of negative words on the emotional polarity of the text was considered. The emotional tendency value of the review text was weighted and calculated, and this was the user’s evaluation value for each index. The specific implementation framework is shown in Figure 3:

#### 3.3.1. Building an Emotional Lexicon

Emotion words are words with an obvious emotional tendency, such as “happy” and “depressed”, to express mood. The collection of emotion words constitutes the emotion lexicon. For the text characteristics of online comments of mobile medical users, we mainly used the set of sentiment analysis words from the China National Knowledge Infrastructure (CNKI) and combined the proprietary words in the field of medical and health care with online buzzwords to build positive and negative dictionaries for online comments of mobile medical apps. After the construction of the sentiment dictionary, the polarity of the corresponding sentiment words in users’ online text comments was extracted using the sentiment dictionary. If a word did not correspond to a corresponding sentiment word in the sentiment dictionary, its polarity was determined using a manual approach and the word was added to the corresponding sentiment dictionary. In order to facilitate the processing, the weights of all positive words in the sentiment dictionary were set to 1 and negative words were set to −1.

#### 3.3.2. Degree Adverb Processing

Some words in the review text did not have a sentiment tendency, but they played a role in modifying the sentiment words in the review, significantly enhancing the sentiment tendency of the review. For example, in the words “I don’t use it very well” and “the quality is very good”, “good” is the word that expresses the user’s emotional tendency while “very” modifies the emotional word. Adverbs of degree do not carry any emotion in themselves, but they can enhance or diminish the original degree of emotion of an emotive word. The degree of modification of degree adverbs is divided into several levels, from strong to weak, and the degree of influence on emotional words of degree adverbs with different modification strengths is also different. Based on the degree adverb lexicon of CNKI, combined with the degree level division of the degree adverb lexicon of CNKI and previous studies, this study divided the degree adverbs into four levels and assigned appropriate weights to the degree adverbs according to their comments on emotion. The degree adverb weights were assigned as shown in Table 1.

#### 3.3.3. Negative Word Processing

A negative adverb is a type of adverb that has a unique grammatical meaning and impact in the text. In a commentary sentence, if a negative adverb appears, the literal meaning of the sentence and the intended meaning are likely to be different, because the emotion word modified by the negation tends to change the emotional polarity. When a negative word modifies a positive emotion word, the originally expressed positive emotion becomes a negative one, and vice versa. Because of the phenomenon of multiple negation in Chinese, when a negative word appears an odd number of times, the statement indicates negation, and when a negative word appears an even number of times, the statement indicates affirmation. Therefore, this study first constructed a negation lexicon. Then, the punctuation marks in the comments were used as the criteria for dividing the sentences. Finally, the original sentiment polarity was reversed when an odd number of negation words appeared in the sentences.

#### 3.3.4. Exclamatory Sentence Processing

Exclamatory sentences are dominated by the expression of feelings and are often identified by the use of exclamation marks at the end of the sentence. Exclamations are dependent on the emotional polarity of the utterance they are in. They are an enhancement of the user’s degree of emotion. In this study, the weight of exclamation sentences containing emotional words was set to increase by two times.

In summary, the emotional tendency value of each attribute could be obtained. In this study, punctuation was used as the segmentation criterion to divide online comment statements, d, into p sentences, and each sentence was extracted to k sentiment words, with $w_{zt}$ indicating the t-th sentiment word in the z-th sentence. Its sentiment propensity value was calculated as follows.

(1) $$O_{w_{i}}={\left(-1\right)}^{\alpha }\times {\left(2\right)}^{\beta }\times S_{w_{zt}}$$

where α indicates the number of negatives, β={0,1} indicates the presence or absence of exclamations, and $S_{w_{zt}}$ indicates the weight of a sentiment word $w_{zt}$. Then, the final sentiment tendency value of online comment statement, $d_{\xi }$, is:

(2) $$O_{d}=\sum_{z=1}^{p}\sum_{t=1}^{k}O_{w_{zt}}$$

### 3.4. Rough Number-Based Attribute Determination

After the construction of the user satisfaction evaluation system for mobile medical apps was completed, relevant experts were invited to evaluate the importance of the results of the topic-clustering analysis. The complexity of the real decision-making environment and limited human cognition exacerbate the ambiguity of information; uncertainty prevails, and decision makers show ambiguity and diversity in the evaluation process [25]. Based on the classical rough set theory, Zhai et al. [26] proposed a rough number theory containing numerical features such as upper and lower rough limits and intervals. The theory does not require additional assumptions or a priori knowledge to process decision makers’ preferred information directly with raw scoring data. This not only reflects the true feelings of the decision maker, but also allows all evaluation information to be considered together, eliminating subjectivity and inaccuracies in the evaluation information.

**Figure 4:**
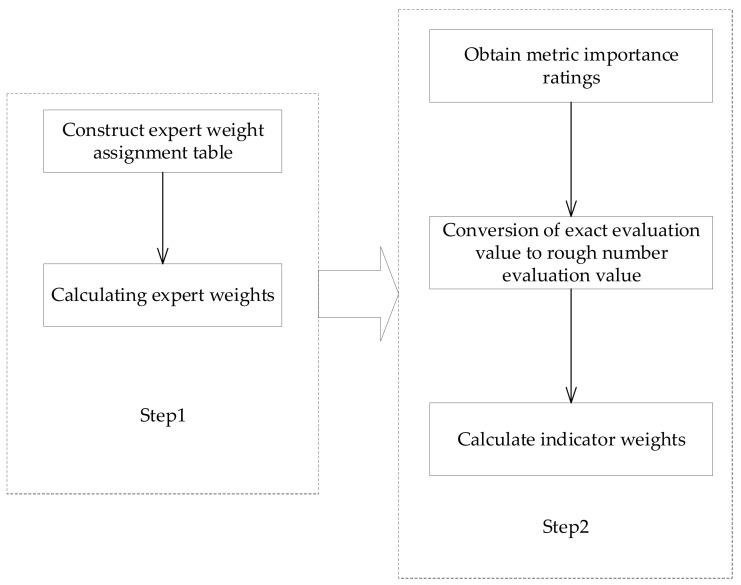
Indicator weights.

Accordingly, to ensure scientific and accurate evaluation results, this study proposes a method for determining attribute weights based on rough numbers, taking full account of information based on the preferences of different experts for different attributes in order to achieve an objective and accurate assessment. The specific implementation framework is shown in Figure 4.

#### 3.4.1. Determining Expert Weights

The differences in personal experiences, occupations, and backgrounds lead to differences in individual thinking, and different individuals may have different perceptions and preferences with respect to the same attribute. Therefore, to ensure scientific and reasonable results, we assigned weights to experts according to the weights’ importance. We constructed an expert weight assignment table based on experts’ occupational relevance, experience, and background knowledge (see Table 2), according to which the conversion rules formed the weight factors of different experts.

According to Table 2, the formula for calculating expert weights was as follows:

(3) $$\delta_{k}=\frac{A_{i}^{k}}{\sum_{k=1}^{m}\sum_{i=1}^{n}A_{i}^{k}}$$

where $\delta_{k}$ denotes the weight of the k-th expert, $A_{i}^{k}$ denotes the weight score of the i-th factor of the k-th expert, n represents the number of expert weight factors, and m denotes the number of experts.

#### 3.4.2. Determining Evaluation Attribute Weights

Experts use 1~10 to assess the importance of each attribute, respectively, with higher scores indicating the more important attributes. Let the importance evaluation of the j-th attribute be expressed as $RI_{j}=\{RI_{j}^{k}\},k=1,2,\ldots ,m$, where $RI_{j}^{k}$ is the importance evaluation value of the k-th expert for the j-th attributes. In order to fully reflect the fuzziness and uncertainty in the expert evaluation process, this study used rough numbers to transform the expert evaluation values. The rough lower approximation $\underline{Apr}(RI_{j}^{k})$ and the rough upper approximation $\bar{Apr}(RI_{j}^{k})$ of the evaluation value $RI_{j}^{k}$ can be expressed as:

(4) $$\underline{Apr}(RI_{j}^{k})=\cup (RI_{j}^{k}\in RI_{j}|RI_{j}^{h}\leq RI_{j}^{k})$$

(5) $$\bar{Apr}(RI_{j}^{k})=\cup (RI_{j}^{k}\in RI_{j}|RI_{j}^{h}\geq RI_{j}^{k})$$

Then, the lower $\underline{\lim}(R_{j}^{k})$ and the upper $\bar{\lim}(R_{j}^{k})$ bounds of $RI_{j}^{k}$, respectively, can be obtained based on the rating values:

(6) $$\underline{\lim}(R_{j}^{k})=[\underline{\lim}(R_{j}^{kL}),\underline{\lim}(R_{j}^{kU})]=[\frac{1}{N_{j}^{L}}\sum_{g=1}^{N_{j}^{L}}RIL_{g}^{L},\frac{1}{N_{j}^{L}}\sum_{g=1}^{N_{j}^{L}}RIL_{g}^{U}]$$

(7) $$\bar{\lim}(R_{j}^{k})=[\bar{\lim}(R_{j}^{kL}),\bar{\lim}(R_{j}^{kU})]=[\frac{1}{N_{j}^{U}}\sum_{g=1}^{N_{j}^{U}}RIL_{g}^{L},\frac{1}{N_{j}^{U}}\sum_{g=1}^{N_{j}^{U}}RIL_{g}^{U}]$$

It can be seen that each factor is defined with its upper and lower limits and consists of interval rough numbers, and the rough evaluation value corresponding to evaluation value $RI_{j}^{k}$ is as follows:

(8) $$RN(RI_{j}^{k})=[\underline{\lim}(RI_{j}^{kL}),\bar{\lim}(RI_{j}^{kU})]=[RI_{j}^{kL},RI_{j}^{kU}]$$

Accordingly, the expert evaluation information is integrated as shown in Equation (7), where $RN\left(RI_{J}^{K}\right)$ denotes the group rough number evaluation value of the j-th attribute.

(9) $$RN(RI_{j})=\sum_{k=1}^{m}\delta_{k}RN(RI_{j}^{k})=[RI_{j}^{L},RI_{j}^{U}]$$

After obtaining the importance rough number evaluation matrix of each attribute, the weight value of each attribute had to be determined. First, the rough number evaluation values were normalized as follows:

(10) $$\left\{\begin{array}{c} \tilde{RI_{j}^{L}}=(RI_{j}^{L}-\underset{j}{\min}(RI_{j}^{L}))/\Delta_{\min}^{\max}\\ \tilde{RI_{j}^{U}}=(RI_{j}^{U}-\underset{j}{\min}(RI_{j}^{U}))/\Delta_{\min}^{\max} \end{array}\right.$$

where $\Delta_{\min}^{\max}\underset{j}{=}\max(RI_{j}^{L})-\underset{j}{\min}(RI_{j}^{L})$, followed by the CFCS (converting the fuzzy data into crisp scores) method to de-fuzzify and convert the rough numbers to exact numbers. The conversion method was as follows.

(1) The conversion factor was defined as:

(11) $$\beta_{j}=\frac{\tilde{RI_{j}^{L}}\times (1-\tilde{RI_{j}^{L}})+\tilde{RI_{j}^{U}}\times \tilde{RI_{j}^{U}}}{1-\tilde{RI_{j}^{L}}+\tilde{RI_{j}^{U}}}$$

(2) The rough numbers were converted to exact numbers to obtain the final fixed values of the evaluated values:

(12) $$\tilde{RF_{j}^{FIN}}=\underset{j}{\min}RI_{j}^{L}+\beta_{i}\Delta_{\min}^{\max}$$

(3) The final weights of each attribute were:

(13) $$\omega_{j}=\frac{RI_{j}}{\sum_{j=1}^{p}RI_{j}}$$

where j=1,2,…,p denotes p attributes, and $\omega_{j}$ denotes the weight value of the j-th attribute and satisfies $\sum_{j=1}^{p}\omega_{j}=1$.

### 3.5. User Satisfaction Measurement Based on Fuzzy Comprehensive Evaluation Method

First, the satisfaction level evaluation set was constructed. Let $V=\left\{V_{1},V_{2},V_{3},V_{4},V_{5}\right\}=\left\{1,2,3,4,5\right\}$, where $V_{5}$ stands for “very satisfied”, $V_{4}$ stands for “satisfied”, $V_{3}$ stands for “average”, $V_{2}$ stands for “unsatisfied “, and $V_{1}$ represents “very dissatisfied”. As in Equation (14):

(14) $$V=\left\{\begin{array}{l} 5;\;O_{d_{i}}\geq 5\\ 4;\;2\leq O_{d_{i}}<5\\ 3;\;-2<O_{d_{i}}<2\\ 2;\;-5<O_{d_{i}}\leq -2\\ 1;\;O_{d_{i}}\leq 5 \end{array}\right.$$

A three-level index evaluation matrix based on emotional disposition values was constructed as follows:

(15) $$R=\left(\begin{array}{ccc} r_{11} & \ldots & r_{15}\\ \vdots & \ddots & \vdots \\ r_{i1} & \ldots & r_{i5} \end{array}\right)$$

where $r_{ij}$ is the affiliation of the i-th evaluation attribute at the j-th satisfaction level, namely, the value of the frequency distribution of the i-th evaluation attribute at the j-th satisfaction level.

(16) $$r_{ij}=\frac{\mathrm{Number}\;\mathrm{of}\;\mathrm{people}\;\mathrm{choosing}\;\mathrm{satisfaction}\;\mathrm{level}\;j\;\mathrm{in}\;\mathrm{the}\;i-\mathrm{th}\;\mathrm{indicator}}{\mathrm{Total}\;\mathrm{number}\;\mathrm{of}\;\mathrm{people}\;\mathrm{involved}\;\mathrm{in}\;\mathrm{the}\;\mathrm{evaluation}\;\mathrm{of}\;\mathrm{the}\;\mathrm{ith}\;\mathrm{indicator}},\;\mathrm{and}\;r_{\mathrm{ij}}\;\in \;[0,1]$$

The combined judgment vector of the three levels of indicators was $B_{i}{}^{c}=w_{i}{}^{c}\times R=(b_{1}^{c},b_{2}^{c},b_{3}^{c},b_{4}^{c},b_{5}^{c})$, where $w_{i}^{c}$ denotes a vector of three-level indicator weights. If $B_{1}+B_{2}+B_{3}+B_{4}+B_{5}\neq 1$, the initial result must be normalized to obtain the final result as $\bar{B_{i}^{c}}=\left(b_{1}^{c}/\sum b_{i}^{c},b_{2}^{c}/\sum b_{i}^{c},b_{3}^{c}/\sum b_{i}^{c},b_{4}^{c}/\sum b_{i}^{c},b_{5}^{c}/\sum b_{i}^{c}\right)$. Synthesis with the evaluation set vector was performed to obtain the fuzzy composite evaluation value of satisfaction, $F^{c}=\bar{B_{i}^{c}}\times V$.

The comprehensive judgment vector of secondary indicators is based on the judgment vector of tertiary indicators, namely:$B_{i}^{b}=w_{i}{}^{b}\times \bar{B_{i}^{c}}=(b_{1}^{b},b_{2}^{b},b_{3}^{b},b_{4}^{b},b_{5}^{b})$. If $B_{1}+B_{2}+B_{3}+B_{4}+B_{5}\neq 1$, the results must be normalized to obtain the final result as $\bar{B_{i}^{b}}=\left(b_{1}^{b}/\sum b_{i}^{b},b_{2}^{b}/\sum b_{i}^{b},b_{3}^{b}/\sum b_{i}^{b},b_{4}^{b}/\sum b_{i}^{b},b_{5}^{b}/\sum b_{i}^{b}\right)$. Synthesis with the evaluation set vector was performed to obtain the fuzzy composite evaluation value of the secondary indicators, $F^{b}=\bar{B_{i}^{b}}\times V$.

Similarly, the integrated judgment vector of the first-level indicators was obtained as $\bar{B_{i}^{a}}=\left(b_{1}^{a}/\sum b_{i}^{a},b_{2}^{a}/\sum b_{i}^{a},b_{3}^{a}/\sum b_{i}^{a},b_{4}^{a}/\sum b_{i}^{a},b_{5}^{a}/\sum b_{i}^{a}\right)$. Finally, the fuzzy composite evaluation value of satisfaction was obtained by synthesizing with the evaluation set vector, $F^{a}=\bar{B_{i}^{a}}\times V$.

## 4. Example Analysis

### 4.1. Data Acquisition and Pre-Processing

In this study, we chose “Huawei App Store” as the data source to study the review data of popular mobile medical apps, and then to discover the factors that affect user satisfaction. First, we collected 2626 reviews by Python, including user nicknames, review times, and review content. Since text data has a lot of noise, the comments that could not be directly analyzed and mined, such as duplicate comments, invalid comments, irrelevant comments, and inadequate information, were deleted. Finally, the remaining 2592 data formed the corpus source for the LDA model. Pre-processing operations, such as word separation and removal of deactivated words, were performed on the corpus source files to obtain the document-word matrix. Then, the LDA model was constructed with the help of the sklearn package in Python software.

### 4.2. Satisfaction Evaluation System Construction

**Figure 5:**
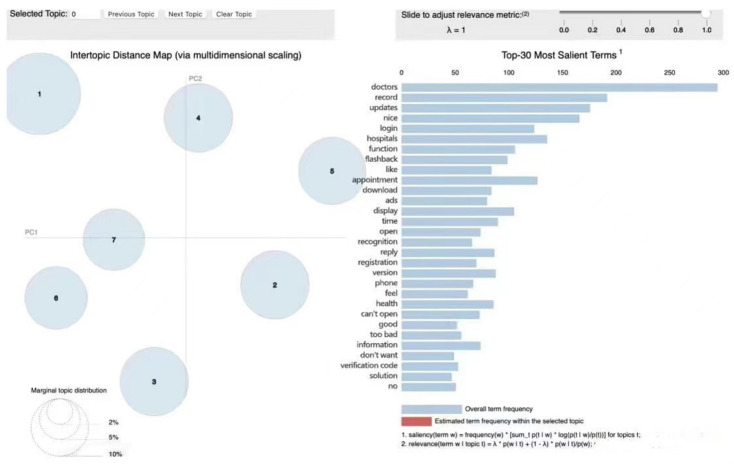
Clustering visualization results of online review topics of mobile medical apps. The circle on the left corresponds to the seven themes, and the words on the right correspond to the topics. The distance between different circles indicates the closeness of different themes. If the circles overlap, there is a crossover between the features of different themes.

First, we set the subject number interval to [0,20] and the step size to 2, with α and β set to default values. We set λ = 1, and the top 10 words with high frequency under each category of topics were taken as the results of topic clustering. Second, the elbow law of model perplexity was used to determine the optimal number of topics for the model, which was 7. The topic clustering results are shown in Table 3, and their visualization results are shown in Figure 5.

According to the grounded theory research method, from practical observation, three-stage coding and theoretical saturation test were conducted on the basis of topic clustering to integrate relevant concepts and attributes, so as to construct a bottom-up evaluation system of user satisfaction with mobile medical apps. The main operation process was as follows: (1) Open-ended coding was used to label the subject terms of the clustering results of the LDA model, to objectively organize the data, and to extract the concepts as the basis for constructing the satisfaction evaluation model for. mobile medical apps. We invited experienced users of mobile medical apps and experts in the healthcare field to categorize the subject terms and analyze their relevance to further summarize their core categories. For example, the topic words “attitude”, “reply”, and “speechless” were summarized as “people interactivity”; the topic words “update”, “download”, and “system” were summarized as “system stability”; and the topic words “doctor”, “professional”, and “good” were summarized as “physician expertise”. Then, 12 core categories were summarized from the 70 subject terms. (2) On the basis of the 12 core categories, the main categories and their relationships were further summarized, using selective coding and a theoretical saturation test. For example, “accuracy and comprehensiveness of information”, “content simplicity”, and “information authenticity” were summarized as “content quality”; “timeliness of response”, “people interactivity”, and “physician expertise” was summarized as “service quality”. Repeating the above three processes, no new categories or concepts were found, and thus it was clear that the model used in this study was theoretically saturated.

**Figure 6:**
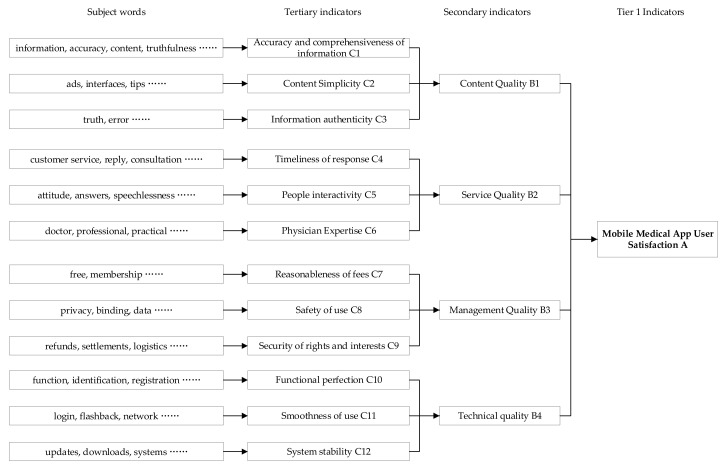
User satisfaction evaluation system for mobile medical apps.

In this study, the 12 core categories are defined as tertiary indicators, including: content simplicity, people interactivity, reasonableness of fees, and functional perfection, etc. And the 4 main categories are defined as secondary indicators, including: content quality, management quality, service quality, and technology quality. Based on this, this study constructs a mobile medical APP user satisfaction evaluation system, as shown in Figure 6.

### 4.3. Satisfaction Assessment

#### 4.3.1. Weighting Calculation

From September to October 2021, four experts in relevant fields, such as mobile communication operations, hospital management, and health development research, were invited to participate in expert consultation. According to the expert weight conversion rules set out in Table 1, the four experts’ weights were assigned in terms of occupational relevance, experience, and background knowledge. The weights were 0.2, 0.25, 0.4, and 0.15, respectively. After that, the four experts assessed the importance of each evaluation attribute using a score of 1~10. For example, the evaluation matrix of the four experts for the secondary indicators was as follows.

$$\left(\begin{array}{cccc} 6 & 6 & 6 & 7\\ 7 & 7 & 6 & 7\\ 6 & 7 & 7 & 8\\ 8 & 7 & 8 & 9 \end{array}\right)$$

To minimize the subjectivity and ambiguity of the experts, the expert evaluation scores were transformed into a rough form. For example, the evaluation score of expert 1 for $B_{4}$ was transformed into a rough number form.

$$\underline{Apr}(8)=\left(8,7,8\right);\;\bar{Apr}(8)=\left(8,8,9\right);\;RI_{B_{4}}^{1}=\left[\left(8+7+8\right)/3,\left(8+8+9\right)/3\right]=\left[7.67,8.33\right]$$

According to Equation (9), the group evaluation indices can be calculated, followed by converting the group rough number evaluation values into definite values according to Equations (10)–(13) and obtaining the weights for each index, as shown in Table 4.

#### 4.3.2. Evaluation Information Acquisition

According to the Chinese wording habits, comment sentences were assigned to the corresponding categories of evaluation indicators at each level, and the comments that did not belong to the evaluation system were deleted. Then, the evaluation information was weighted according to the emotion dictionary, degree adverbs, exclamations, and negatives to determine the emotional tendency value of each indicator, i.e., the value of users’ evaluations of each indicator. Finally, the satisfaction level affiliation of the three-level indicators of mobile medical apps was calculated according to the satisfaction level evaluation set; the results are shown in Table 5.

#### 4.3.3. Satisfaction Measurement

**Figure 7:**
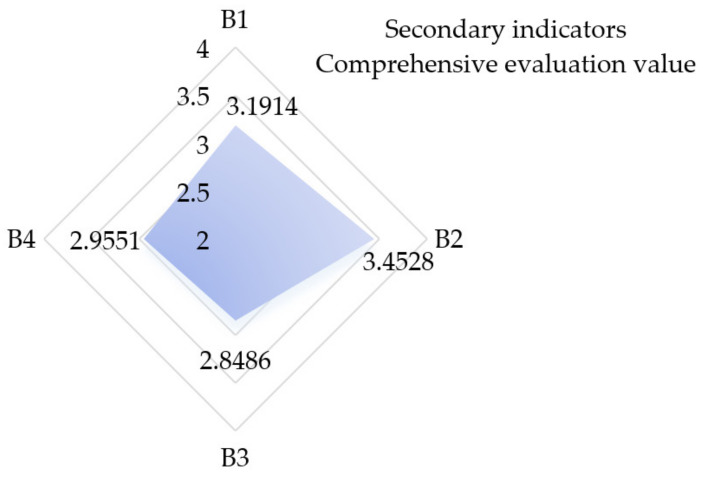
Results of comprehensive evaluation value of secondary indicators.

The fuzzy comprehensive evaluation method is a comprehensive evaluation method based on fuzzy mathematics. It has the characteristics of clear results and strong systematicity, which can be used to better solve the fuzzy and difficult quantification of problems. Therefore, it is suitable for various non-deterministic problems. In order to establish an overall evaluation of satisfactions with mobile medical apps, the fuzzy comprehensive evaluation method was used to calculate the comprehensive evaluation value of secondary indicators; the results are shown in Figure 7.

From the calculation results, it can be seen that the secondary indicators are ranked in the following descending order of satisfaction: service quality, content quality, technical quality, and management quality. In Figure 7, service quality and content quality are ranked between 3 and 4, which means that users are satisfied with the service quality of mobile medical apps; the indicators of management quality and technical quality ranked between 2 and 3, which means that users are not very satisfied with all these indicators. Finally, based on the evaluation value of secondary indicators, the total score of user satisfaction was calculated as 3.0100, which is between general satisfaction and satisfaction that is close to general satisfaction, indicating that users are generally satisfied with mobile medical apps.

## 5. Discussion

First, the results of the thematic analysis show that users of mobile medical apps are concerned about 12 elements, including the timeliness of responses and the reasonableness of fees, which means that these 12 elements have a great impact on user satisfaction. According to the calculation results in ranking the weight of the three levels of indicators, we found that compared to the other indicators, functional perfection had the highest weight. This is probably due to the fact that functional perfection provides good support for users in their search for medicine and can better meet their needs; therefore, functional perfection was the most important indicator. System stability, smoothness of use, and accuracy and comprehensiveness of information were also given higher weights. This is probably because a stable system and smooth usage enhance the user’s experience, generating positive emotions and inspiring the user to continue using the app, while accurate and comprehensive information arouses a user’s desire to read, further increasing the perceived satisfaction. Therefore, in the development of mobile medical apps, special attention must be paid to functional perfection, system stability, smoothness of use, and accurate and comprehensive information.

Second, on the basis of these 12 elements, this study summarized four secondary indicators: service quality, content quality, technical quality, and management quality. Based on the results of the indicator weight calculation, the weights of the four secondary indicators were relatively close, with technical quality having the highest weight, i.e., the highest importance, followed by management quality, service quality, and content quality. This is probably due to the fact that, compared to the other three indicators, good technical quality increases the perceived ease of use of the platform, which in turn influences users’ willingness to use the platform and their satisfaction in doing so. Accordingly, users are more concerned about the technical quality of the platform.

The results of studying user satisfaction with mobile medical apps show that users are generally satisfied with mobile medical apps, and that mobile medical apps need further improvement. In addition, by comparing the comprehensive evaluation values of secondary indicators, we found that users’ satisfaction with management quality and technical quality is low, compared to their satisfaction with service quality and content quality. This may be due to the fact that, at present, the regulatory power of the platforms is not sufficient, and some of the platforms do not have detailed fee standards or standardized refund processes. At the same time, during the use of the system, there were frequent flashbacks, black screens, and incompatibility problems that greatly reduced users’ senses of their experiences and led to poor user satisfaction. Therefore, more attention should be paid to the management quality and technical quality of mobile medical apps in the future.

Based on the above research, this study provides some valuable insights for managers of mobile medical apps, as follows (1) Security supervision can be strengthened and the quality of management can be enhanced. On the one hand, the platform should provide for developing a reasonable pricing mechanism on the basis of adequate research by strictly following the charging standards and showing the charging items to users in a timely manner to prevent the emergence of duplicate and indiscriminate charges. At the same time, the platform needs to improve its operating model by not excessively collecting user information and protecting user privacy. In addition, the service process should be optimized (especially the refund process) to protect the legal rights of users, reduce perceived risks, and enhance perceived utility [27]. (2) The platform should increase investment in system construction, improve system functions such as system stability and compatibility, prevent problems such as flashback, black screen, and incompatibility, and enhance system quality, so as to enhance users’ perception of the ease of use of mobile medical apps and improve their satisfaction and willingness to use these apps. (3) Information auditing can be strengthened to ensure the quality of content. The platform should improve the authenticity and accuracy of content by strengthening the auditing of published content, improving the reliability of content by strictly vetting the qualifications of practitioners, and enhancing the comprehensiveness of content by real-time updates. (4) Access to doctors can be strengthened by an emphasis on services and their quality management. Mobile medical apps can be incorporated into the performance evaluation system of hospitals, and quality mobile medical services can be included in medical staff job descriptions, performance appraisals, and other aspects related to the development of medical staff. Administrative staff should formulate corresponding incentive policies to ensure that the values of medical staff contributions are recognized, so as to improve doctors’ satisfaction and initiative.

## 6. Conclusions

First, this study adopted online comments as the data source and used the LDA topic model to obtain, from users’ online comments, the influencing factors for users’ attentions. Based on the three-stage coding of ground theory and the theoretical saturation test, we constructed a bottom-up evaluation system for user satisfaction with mobile medical apps, including 12 indicators in four dimensions: content quality, service quality, management quality, and technical quality. Next, information on users’ evaluations of each indicator was obtained by calculating the sentiment tendency values in the online reviews. Then, in order to fully consider the fuzziness and uncertainty in the expert decision-making process and the influence of weights on the decision results, we formulated expert weight conversion rules and processed expert evaluation information with the help of rough numbers to determine the weight of experts and evaluation indicators, so as to improve the accuracy of the evaluation results. Finally, the fuzzy comprehensive evaluation method was used to achieve a quantitative evaluation of user satisfaction with mobile medical apps. This study provides the following contributions:

(1) With the development of science and technology, users’ satisfaction with mobile medical apps is changing faster and faster, placing higher demands on the product market response. The ability of managers to keep abreast of changes in user satisfaction has become the key to developing mobile medical apps. Online reviews, as a form of word-of-mouth reviews, have become a major channel for users to discuss products and services with each other, to obtain information, and to express their needs. Therefore, this study examined user satisfaction based on online reviews, which was conducive to understanding and reflecting on the use of mobile medical apps and promoting the sustainable development of mobile medicine.

(2) We proposed that the model for user satisfaction with mobile medical apps can capture the influencing factors and the degree of implementation of user concerns, taking into account the influence of experts and indicator weights on decision results as well as the ambiguity and uncertainty of linguistic information. This model is innovative not only in regard to user satisfaction evaluation, but it also has certain reference value for updating and improving mobile medical apps and operation management.

This study analyzed online reviews to understand users’ expressed needs and to help managers understand users’ preferences; however, there are still shortcomings. This study was not able to effectively screen out false comments in processing user comment text, and more in-depth research will be conducted in this area in the future.

## Figures and Tables

**Table 1:** Adverb dictionary.

Degree Level	Adverbs of Degree	Weighting
Extremely	entirely, completely, absolutely, extremely......	2
High	quite a few, special, really, extra, too......	1.75
Medium	more, more and more, rather, increasingly, far......	1.5
Low	slightly, a little, half a point, a few, small......	0.5

**Table 2:** Expert weight distribution table.

Factors	Levels	Score
Occupation Relevance	High	4
	Medium-high	3
	Low to medium	2
	Low	1
Experience	10 or more	4
	5~9 years	3
	2~5 years	2
	Less than 2 years	1
Background Knowledge	PhD	4
	Master	3
	Undergraduate	2
	High School	1

**Table 3:** Top 7 topics and subject terms for online reviews of mobile medical apps.

Total Number of Documents	Number of Supported Documents	Topics
2592	524	Time; Recognition; Interface; Customer; Service; Don’t want; Practical; Solution; Speechless; Report; Good; review
	368	Flashback; Appointment; Registration; Phone; Information; Verification code; Not up to; Sign; No Binding
	368	Login; Show; Ads; Download; Open; Too bad; Tip; Network; Error; Failed
	368	Updates; Versions; Health; Appointments; System; Logistics; Vaccines; Recommendations; Members; Refunds
	358	Doctors; Hospitals; Reply; Professional; Ask a question; Consultations; Platform Register; Free; Attitude
	308	Like; Feel; Good; Can’t open; Accurate; Answer; Content; Satisfaction; Version; Privacy
	298	Record; Function; Data; Nice; Display; Member; Detail; Inoculation; Real; Great

**Table 4:** Expert weighting results.

Evaluation Properties	Experts				Group Assessment Value	Standardization	Precise Weights
	$Q_{1}$	$Q_{2}$	$Q_{3}$	$Q_{4}$			
$C_{1}$	[8,8.25]	[8,8.25]	[8,8.25]	[8.25,9]	[8.0375,8.3625]	[0.896,1]	0.41
$C_{2}$	[6,6.75]	[6.75,7]	[6.75,7]	[6.75,7]	[6.6,6.95]	[0.438,0.55]	0.33
$C_{3}$	[5.5,6]	[5.5,6]	[5,5.5]	[5,5.5]	[5.225,5.725]	[0,0.159]	0.26
$C_{4}$	[6,6.75]	[6.5,7.25]	[6.25,7]	[6.75,7.5]	[6.3375,7.0875]	[0.546,1]	0.36
$C_{5}$	[5,5.75]	[5.34,6]	[5.75,7]	[5.34,6]	[5.436,6.35]	[0,0.553]	0.30
$C_{6}$	[6,6.375]	[6,6.375]	[6.16,6.75]	[6.375,7]	[6.12025,6.61875]	[0.414,0.716]	0.34
$C_{7}$	[6,6.25]	[6,6.25]	[6.25,7]	[6,6.25]	[6.1,6.55]	[0,0.242]	0.29
$C_{8}$	[7.75,8]	[7.75, 8]	[7.75,8]	[7,7.75]	[7.6375,7.9625]	[0.826,1]	0.36
$C_{9}$	[6.75,7.67]	[7.375,8]	[7.375,8]	[6.5,7.375]	[7.11875,7.84025]	[0.547,0.934]	0.35
$C_{10}$	[6,6.25]	[6,6.25]	[6.25,6.5]	[6.25,6.5]	[6.1375,6.3875]	[0.73,1]	0.35
$C_{11}$	[5,5.75]	[5.5,6.25]	[5.75,6.5]	[5.25,6]	[5.4625,6.2125]	[0,0.811]	0.32
$C_{12}$	[5.83,6.17]	[5.5,6]	[6,6.5]	[5.83,6.17]	[5.8155,6.2595]	[0.382,0.862]	0.33
$B_{1}$	[6,6.25]	[6,6.25]	[6,6.25]	[6.25,7]	[6.0375,6.3625]	[0,0.141]	0.22
$B_{2}$	[6.75,7]	[6.75,7]	[6,6.75]	[6.75,7]	[6.45,6.9]	[0.179,0.373]	0.24
$B_{3}$	[6,7]	[6.67,7.33]	[6.67,7.33]	[7,8]	[6.5855,7.3645]	[0.237,0.574]	0.25
$B_{4}$	[7.67,8.33]	[7,8]	[7.67,8.33]	[8,9]	[7.552,8.348]	[0.655,1]	0.29

**Table 5:** User satisfaction level affiliation for mobile medical apps.

	Very Satisfied	Satisfaction	General	Dissatisfaction	Very Dissatisfied
$C_{1}$	16 (0.12)	27 (0.21)	71 (0.55)	13 (0.1)	3 (0.02)
$C_{2}$	7 (0.09)	17 (0.21)	30 (0.37)	21 (0.26)	6 (0.07)
$C_{3}$	16 (0.14)	23 (0.2)	55 (0.47)	19 (0.16)	3 (0.03)
$C_{4}$	30 (0.12)	68 (0.26)	118 (0.46)	38 (0.15)	5 (0.02)
$C_{5}$	35 (0.24)	36 (0.24)	47 (0.32)	22 (0.15)	7 (0.05)
$C_{6}$	24 (0.24)	26 (0.26)	37 (0.38)	9 (0.09)	3 (0.03)
$C_{7}$	13 (0.09)	37 (0.27)	67 (0.48)	20 (0.14)	2 (0.02)
$C_{8}$	1 (0.03)	6 (0.15)	17 (0.44)	13 (0.33)	2 (0.05)
$C_{9}$	4 (0.04)	8 (0.09)	32 (0.34)	44 (0.46)	7 (0.07)
$C_{10}$	32 (0.08)	77 (0.18)	237 (0.56)	63 (0.15)	12 (0.03)
$C_{11}$	9 (0.03)	19 (0.07)	183 (0.62)	68 (0.23)	16 (0.05)
$C_{12}$	8 (0.03)	33 (0.1)	217 (0.67)	52 (0.16)	12 (0.04)

## Data Availability

Data sharing is not applicable to this article.
